# A diagnostic model for coronavirus disease 2019 (COVID-19) based on radiological semantic and clinical features: a multi-center study

**DOI:** 10.1007/s00330-020-06829-2

**Published:** 2020-04-16

**Authors:** Xiaofeng Chen, Yanyan Tang, Yongkang Mo, Shengkai Li, Daiying Lin, Zhijian Yang, Zhiqi Yang, Hongfu Sun, Jinming Qiu, Yuting Liao, Jianning Xiao, Xiangguang Chen, Xianheng Wu, Renhua Wu, Zhuozhi Dai

**Affiliations:** 1 Department of Radiology, Meizhou People’s Hospital, Meizhou, 514031 Guangdong People’s Republic of China; 2 Department of Radiology, 2nd Affiliated Hospital, Shantou University Medical College, Shantou, 515000 Guangdong People’s Republic of China; 3 Department of Radiology, First Affiliated Hospital, Shantou University Medical College, Shantou, 515041 Guangdong People’s Republic of China; 4 Department of Radiology, Huizhou Municipal Central Hospital, Huizhou, 516001 Guangdong People’s Republic of China; 5 Department of Radiology, Shantou Central Hospital, Shantou, 515041 Guangdong People’s Republic of China; 6 Department of Radiology, Yongzhou People’s Hospital, Yongzhou, 425006 Hunan People’s Republic of China; 7 School of Information Technology and Electrical Engineering, University of Queensland, Brisbane, Queensland 4072 Australia; 8 GE Healthcare, Guangzhou, 510623 People’s Republic of China; 9 Provincial Key Laboratory of Medical Molecular Imaging, Shantou, Guangdong People’s Republic of China

**Keywords:** COVID-19, Pneumonia, Radiology, Diagnosis, Multi-institutional systems

## Abstract

**Objectives:**

Rapid and accurate diagnosis of coronavirus disease 2019 (COVID-19) is critical during the epidemic. We aim to identify differences in CT imaging and clinical manifestations between pneumonia patients with and without COVID-19, and to develop and validate a diagnostic model for COVID-19 based on radiological semantic and clinical features alone.

**Methods:**

A consecutive cohort of 70 COVID-19 and 66 non-COVID-19 pneumonia patients were retrospectively recruited from five institutions. Patients were divided into primary (*n* = 98) and validation (*n* = 38) cohorts. The chi-square test, Student’s *t* test, and Kruskal-Wallis *H* test were performed, comparing 1745 lesions and 67 features in the two groups. Three models were constructed using radiological semantic and clinical features through multivariate logistic regression. Diagnostic efficacies of developed models were quantified by receiver operating characteristic curve. Clinical usage was evaluated by decision curve analysis and nomogram.

**Results:**

Eighteen radiological semantic features and seventeen clinical features were identified to be significantly different. Besides ground-glass opacities (*p = 0.032*) and consolidation (*p = 0.001*) in the lung periphery, the lesion size (1–3 cm) is also significant for the diagnosis of COVID-19 (*p = 0.027*). Lung score presents no significant difference (*p = 0.417*). Three diagnostic models achieved an area under the curve value as high as 0.986 (95% CI 0.966~1.000). The clinical and radiological semantic models provided a better diagnostic performance and more considerable net benefits.

**Conclusions:**

Based on CT imaging and clinical manifestations alone, the pneumonia patients with and without COVID-19 can be distinguished. A model composed of radiological semantic and clinical features has an excellent performance for the diagnosis of COVID-19.

**Key Points:**

*• Based on CT imaging and clinical manifestations alone, the pneumonia patients with and without COVID-19 can be distinguished.*

*• A diagnostic model for COVID-19 was developed and validated using radiological semantic and clinical features, which had an area under the curve value of 0.986 (95% CI 0.966~1.000) and 0.936 (95% CI 0.866~1.000) in the primary and validation cohorts, respectively.*

**Electronic supplementary material:**

The online version of this article (10.1007/s00330-020-06829-2) contains supplementary material, which is available to authorized users.

## Introduction

On January 30, 2020, the World Health Organization (WHO) has declared the severe acute respiratory syndrome coronavirus 2 (SARS-CoV-2) outbreak as a global health emergency of international concern. This outbreak has infected all provinces of China and rapidly spread to the rest of the world. At the time of writing this article (March 16, 2020), there have been more than 158 countries and territories affected [1]. Whole-genome sequencing and phylogenetic analysis reveal that the severe acute respiratory syndrome coronavirus 2 (SARS-CoV-2) is similar to some beta coronaviruses detected in bats, but it is distinct from severe acute respiratory syndrome coronavirus (SARS-Cov) and Middle East respiratory syndrome coronavirus (MERS-CoV) [2].

Patients with COVID-19 develop pneumonia with associated symptoms of fever (98%), cough (76%), and myalgia or fatigue (44%) [3]. CT imaging plays a critical role in the diagnosis and the monitoring of disease progression [4–6]. The latest research studies described the characteristic imaging manifestations of COVID-19, including ground-glass opacities (GGO) (57 to 88%), bilateral involvement (76 to 88%), and peripheral distribution (33 to 85%) [7–10]. Other imaging features such as consolidation, cavitation, and interlobular septal thickening are also reported in some patients [11–13]. However, these imaging manifestations of COVID-19 are nonspecific and are difficult to distinguish from other pneumonia. To our knowledge, there have been no studies explicitly comparing imaging and clinical characteristics between pneumonia patients with and without COVID-19.

The current diagnostic criterion for COVID-19 is the positive result of a nucleic acid test by real-time reverse transcription polymerase chain reaction (RT-PCR) or next-generation sequencing [14]. However, false-negative results caused by unstable specimen processing are relatively high in clinical practice, which has worsened the spread of the outbreak [15–18]. Moreover, laboratory testing for SARS-CoV-2 requires a rigorous platform, which is not assembled in all hospitals. Thus, this requires specimen transfer, which may delay diagnosis for days. Early and accurate diagnosis is crucial, particularly for critically ill patients who need emergency surgery, and with pneumonia complications. To solve these problems, we hypothesize that a diagnostic model can be developed based on CT imaging and clinical manifestations alone, independent of the nucleic acid test.

In this study, we identify the differences in imaging and clinical manifestations between patients with and without COVID-19. We also develop and validate a model for COVID-19 diagnosis based on radiological semantic and clinical features.

## Patients and methods

### Patients

Ethical approvals by the institutional review boards were obtained for this retrospective analysis, and the need to obtain informed consent was waived.

From January 1 to February 8, 2020, seventy consecutive patients with COVID-19 admitted in 5 independent hospitals from 4 cities were enrolled in this study (mean age, 42.9 years; range, 16–69 years), including 41 men (mean age, 41.8 years; range, 16–69 years) and 29 women (mean age, 44.5 years; range, 16–66 years). All patients were confirmed with SARS-CoV-2 infection by real-time RT-PCR and next-generation sequencing. Of these patients, 24 were from Huizhou City, 25 from Shantou City, 15 from Yongzhou City, and the rest 6 from Meizhou City. At the same period, another 66 pneumonia patients without COVID-19 from Meizhou People’s Hospital were recruited as controls (mean age, 46.7 years; range, 0.3–93 years), including 43 men (mean age, 46.0 years; range, 0.3–93 years) and 23 women (mean age, 48.0 years; range, 1–86 years). All the controls were confirmed with consecutive negative RT-PCR assays. Figure E1 in the Supplementary Material shows the patient recruitment pathway for the control group, along with the inclusion and exclusion criteria.

According to previous studies [19–21], whose sample size is comparable with ours, the ratio between primary and validation cohort is 7:3. In this study, a total of 136 patients were divided into primary (*n* = 98) and validation (*n* = 38) cohorts, close to 7:3. A total of 19 COVID-19 patients from two hospitals (6 patients from Meizhou People’s Hospital and 13 patients from the First Affiliated Hospital of Shantou University Medical College) and 19 randomly selected controls from Meizhou City were incorporated into the validation cohort. The rest of the patients are incorporated in the primary cohort, including 51 COVID-19 patients from Huizhou, Yongzhou, and Shantou cities and 47 controls from Meizhou City. The primary cohort was utilized to select the most valuable features and build the predictive model, and the validation cohort was used to evaluate and validate the performance of the model.

### Image and clinical data collection

The chest CT imaging data without contrast material enhancement were obtained from multiple hospitals with different CT systems, including GE CT Discovery 750 HD (General Electric Company), SCENARIA 64 CT (Hitachi Medical), Philips Ingenuity CT (PHILIPS), and Siemens SOMATOM Definition AS (Siemens). All images were reconstructed into 1-mm slices with a slice gap of 0.8 mm. Detailed acquisition parameters were summarized in the Supplementary Material (Table E1).

The clinical history, nursing records, and laboratory findings were reviewed for all patients. Clinical characteristics, including demographic information, daily body temperature, blood pressure, heart rate, clinical symptoms, and history of exposure to epidemic centers, were collected. Total white blood cell (WBC) counts, lymphocyte counts, ratio of lymphocyte, neutrophil count, ratio of neutrophil, procalcitonin (PCT), C-reactive protein level (CRP), and erythrocyte sedimentation rate (ESR) were measured. All threshold values chosen for laboratory metrics were based on the normal ranges set by each individual hospital.

### Image analysis

**Fig. 1:**
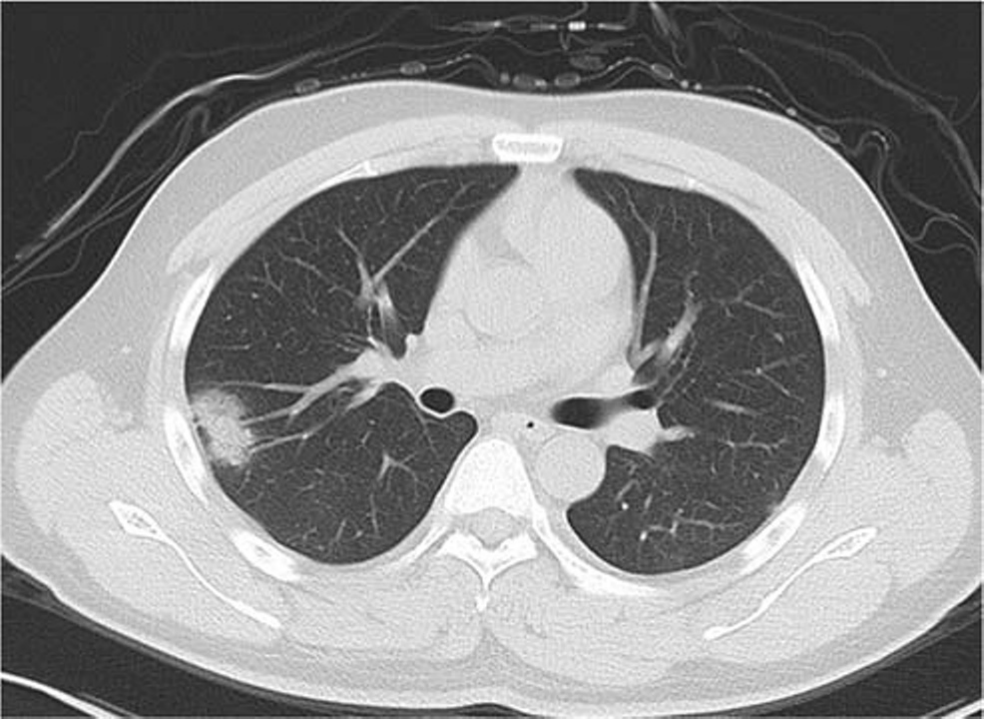
A 23-year-old female with a travel history to Wuhan presenting with fever. Axial noncontrast CT image shows a consolidation with ground-glass opacities in the peripheral region by the right upper lobe. Air bronchogram is found in lesion. The maximum diameter of lesion is 2.8 cm. The right upper lobe score is 1 because of the involved lung parenchyma less than 1/4

For extraction of radiological semantic features, two senior radiologists (D.L. and X.C., more than 15 years of experience) reached a consensus, blinded to clinical and laboratory findings. The radiological semantic features included both qualitative and quantitative imaging features. The lesions in the outer third of the lung were defined as peripheral, and lesions in the inner two-thirds of the lung were defined as central [22]. The progression of COVID-19 lesions within each lung lobe was evaluated by scoring each lobe from 0 to 4 [7], corresponding to normal, 1~25% infection, 26~50% infection, 51~75% infection, and more than 75% infection, respectively. The scores were combined for all five lobes to provide a total score ranging from 0 to 20. A total of 41 radiological features (26 quantitative and 15 qualitative) were extracted for the analysis. The descriptions of radiological semantic features are listed in the Supplementary Material (Table E2). Figure 1 is one example of the evaluation of CT imaging.

### Clinical and radiological feature selection

To obtain the most valuable clinical and radiological semantic features, statistical analysis, univariate analysis, and the least absolute shrinkage and selection operator (LASSO) method were performed. In statistical analysis, the chi-square test, the Kruskal-Wallis *H* test, and *t* test were utilized to compare the radiological semantic and clinical features between COVID-19 and non-COVID-19 groups. The features with *p* value smaller than 0.05 were selected. Then, univariate analysis was performed for clinical and radiological candidate features to determine the COVID-19 risk factors. The features with *p* value smaller than 0.05 in univariate analysis were also selected. The least absolute shrinkage and selection operator (LASSO) method [23] was utilized to select the most useful features with penalty parameter tuning that was conducted by 10-fold cross-validation based on minimum criteria. Diagnostic models were then constructed by multivariate logistic regression with the selected features. The flowchart of the feature selection process for these models was presented in the Supplementary Material (Fig. E2).

### Development and validation of the diagnostic model

**Fig. 2:**
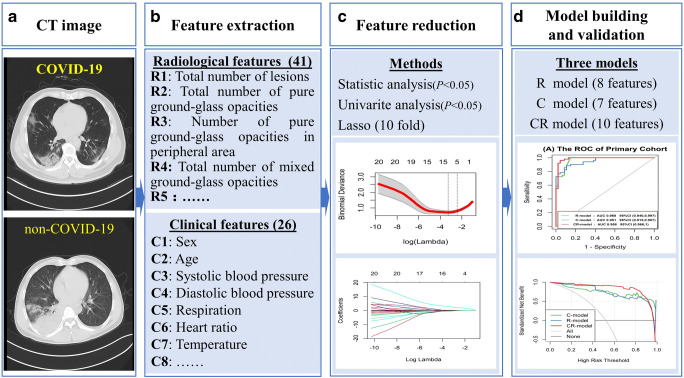
Workflow of data process and analysis in this study. Radiological semantic features, including qualitative and quantitative imaging features, are extracted from axial lung CT section. The clinical manifestation and laboratory parameters are provided by electronic case system. Statistical analysis is performed for comparing the different features between COVID-19 and non-COVID-19 patients. Univariate analysis, least absolute shrinkage, and selection operator (LASSO) are further performed to determine the COVID-19 risk factors with *p* < 0.05 in statistical analysis. Three models based on the selected features are established by multivariate logistic regression. These models include radiological mode (R model), clinical model (C model), and the combination of clinical and radiological model (CR model). The performance and clinical benefits of the prediction model are assessed by the area under a receiver operating characteristic (ROC) curve and the decision curve, respectively

To develop an optimal model, we evaluated 3 models by analyzing (i) the clinical features model (C model), (ii) radiological semantic features model (R model), and (iii) the combination of clinical and radiological semantic features model (CR model) by multivariate logistic regression analysis. The classification performances of the models were evaluated by the area under the receiver operating characteristic (ROC) curve. The area under the curve (AUC), accuracy, sensitivity, and specificity were also calculated. A decision curve analysis was conducted to determine the clinical usefulness of the diagnostic model by quantifying the net benefits at different threshold probabilities in the validation dataset [24]. The development of decision curve was described in the Supplementary Materials. Figure 2 depicts the flowchart of the proposed analysis pipeline described above. We also built a nomogram, which was a quantitative tool to predict the individual probability of infection by COVID-19, based on the multivariate logistic analysis of the CR model with the primary cohort. Depending on the coefficient of the predictive factors in multivariate logistic regression model, all values of each predictive factor were assigned points. A total point was obtained by summing all the points of each predictive factor. The scale also showed the relationship between the total point and the prediction probability in the nomogram. The corresponding calibration curves of the CR model in the primary cohort and validation cohort are shown in the Supplementary Material (Fig. E3).

### Statistical analysis

Statistical analysis was conducted with R software (Version: 3.6.4, http: www.r-project.org/). The reported significance levels were all two-sided, and the statistical significance level was set to 0.05. The multivariate logistic regression analysis was performed with the “stats” package. Nomogram construction was performed using the “rms” package. Decision curve analysis was performed using the “dca. R” package.

## Results

### Imaging and clinical manifestations between groups

The differences between patients with and without COVID-19 for all 67 features (41 imaging and 26 critical clinical features) are shown in Tables 1 and 2 and the Supplementary Materials (Tables E3 and E4). The differences between the primary cohort and validation cohort for the same features are shown in the Supplementary Materials (Tables E5 and E6). All characteristics except fatigue and white blood cell count in the CR model presented no significant difference between the primary and validation cohorts. A total of 1745 lesions were identified, with 1062 from the COVID-19 group and 683 from the non-COVID-19 group.

**Table 1:** Radiological semantic features of patients in COVID-19 and non-COVID-19

Feature	Non-COVID-19 (*n* = 66)	COVID-19 (*n* = 70)	*p* value
Number of pure GGO			
Total^#^	1.00 (0.00, 5.05)	3.50 (0.95, 8.05)	0.018^b^*
Peripheral area^#^	1.00 (0.00, 4.05)	2.00 (0.00, 6.05)	0.032^b^*
Central/both peripheral and central area^#^	0.00 (0.00, 0.00)	0.00 (0.00, 2.00)	0.001^b^*
Number of mixed GGO			
Total^#^	1.00 (0.00, 3.05)	3.00 (1.00, 9.00)	0.001^b^*
Peripheral area^#^	0.00 (0.00, 2.00)	2.50 (1.00, 6.00)	< 0.001^b^*
Central/both peripheral and central area^#^	0.00 (0.00, 1.05)	0.00 (0.00, 2.00)	0.657^b^
Total number of consolidation			
Consolidation^#^	1.00 (0.00, 3.00)	0.00 (0.00, 0.05)	0.001^b^*
Pure solid nodules^#^	0.00 (0.00, 0.00)	0.00 (0.00, 0.00)	0.309^b^
Solid nodules with GGO^#^	0.00 (0.00, 0.00)	0.00 (0.00, 1.00)	0.033^b^*
Total number of lesions			
Peripheral area^#^	5.00 (2.00, 9.05)	7.00 (2.00, 13.00)	0.112^b^
Central area^#^	0.00 (0.00, 3.00)	0.00 (0.00, 1.05)	0.960^b^
Both peripheral and central area^#^	0.00 (0.00, 2.00)	0.00 (0.00, 2.05)	0.582^b^
Interlobular septal thickening			0.009^a^*
Negative	44 (66.67%)	31 (44.29%)	
Positive	22 (33.33%)	39 (55.71%)	
Crazy paving pattern			< 0.001^a^*
Negative	60 (90.91%)	32 (45.71%)	
Positive	6 (9.09%)	38 (54.29%)	
Tree-in-bud sign			< 0.001^a^*
Negative	37 (56.06%)	61 (87.14%)	
Positive	29 (43.94%)	9 (12.86%)	
Pleural thickening			0.030^a^*
Negative	46 (69.70%)	36 (51.43%)	
Positive	20 (30.30%)	34 (48.57%)	
Offending vessel augmentation in lesions			< 0.001^a^*
Negative	55 (83.33%)	17 (24.29%)	
Positive	11 (16.67%)	53 (75.71%)	

*GGO* ground-glass opacities

^#^Results are median with interquartile range in parentheses, and the remainder results are measurements with corresponding ratio in parentheses

*Data with statistical significance. *p*^a^: chi-square test, *p*^b^: Student’s *t* test

**Table 2:** Clinical features of patients in COVID-19 and non-COVID-19

Feature	Non-COVID-19 (*n* = 66)	COVID-19 (*n* = 70)	*p* value
Sex			
Male^#^	43 (65.15%)	41 (58.57%)	0.430^a^
Female^#^	23 (34.85%)	29 (41.43%)	
Age (years)	46.73 ± 25.00	42.93 ± 13.32	0.275^b^
Vital signs			
Systolic blood pressure (mmHg)	126.92 ± 23.07	127.07 ± 15.16	0.965^b^
Diastolic blood pressure (mmHg)	77.74 ± 15.72	80.39 ± 10.51	0.254^b^
Respiration rate (bpm)	25.20 ± 7.29	19.86 ± 1.90	< 0.001^b^*
Heart rate (bpm)	101.59 ± 20.36	86.06 ± 13.34	< 0.001^b^*
Temperature (°C)	37.61 ± 1.06	37.12 ± 0.83	0.003^b^*
Signs			
Dry cough^#^	56 (84.85%)	48 (68.57%)	0.025^a^*
Fatigue^#^	8 (12.12%)	22 (31.43%)	0.007^a^*
Sore throat^#^	6 (9.09%)	9 (12.86%)	0.483^a^
Stuffy^#^	4 (6.06%)	2 (2.86%)	0.623^a^
Runny nose^#^	3 (4.55%)	3 (4.29%)	0.731^a^
White blood cell count (× 10^9^/L)	11.48 ± 5.36	5.27 ± 2.33	< 0.001^b^*
White blood cell count category			< 0.001^c^*
Low^#^	0 (0.00%)	2 (2.86%)	
Normal^#^	27 (40.91%)	63 (90.00%)	
High^#^	39 (59.09%)	5 (7.14%)	
Lymphocyte count (× 10^9^/L)	1.57 ± 1.33	1.25 ± 0.68	0.086^b^
Lymphocyte count category			< 0.001^c^*
Low^#^	24 (36.36%)	32 (45.71%)	
Normal^#^	35 (53.03%)	37 (52.86%)	
High^#^	7 (10.61%)	1 (1.43%)	
Neutrophil count (× 10^9^/L)	8.97 ± 4.90	3.53 ± 2.17	< 0.001^b^*
Neutrophil count category			< 0.001^c^*
Low^#^	3 (4.55%)	8 (11.43%)	
Normal^#^	23 (34.85%)	59 (84.29%)	
High^#^	40 (60.61%)	3 (4.29%)	
C-reactive protein (mg/L)	69.30 ± 65.88	26.37 ± 30.97	< 0.001^b^*
Procalcitonin (ng/mL)	3.36 ± 8.98	0.26 ± 0.84	0.007^b^*

*Data with statistical significance. *p*^a^: chi-square test, *p*^b^: Student’s *t* test. *p*^c^: Kruskal-Wallis *H* test

^#^Results are measurements with corresponding ratio in parentheses

For imaging manifestations, 7 patients in the COVID-19 group showed normal chest CT (10%). COVID-19 patients have a greater number of pure GGO and mixed GGO than non-COVID-19 patients (*p = 0.018* and *p = 0.001*, respectively). For pure GGO lesions, the differences are significant both in peripheral (*p = 0.032*) and in central areas (*p = 0.001*). However, the number of mixed GGO is mainly distributed at the periphery in COVID-19 patients (*p < 0.001*), with no statistical difference in the central area. The consolidation lesions without GGO occurred less in COVID-19 patients (*p = 0.001*). More lesions are between 1 and 3 cm (*p = 0.027*), and fewer lesions are larger than half of the lung segment (*p = 0.017*) in COVID-19 patients. Other significant differences between the two groups include the pleural traction sign (*p = 0.019*), bronchial wall thickening (*p < 0.001*), interlobular septal thickening (*p = 0.009*), crazy paving (*p < 0.001*), tree-in-bud (*p < 0.001*), pleural effusions (*p < 0.001*), pleural thickening (*p = 0.030*), and the offending vessel augmentation in lesions (*p < 0.001*). The lung score presents no significant difference between the COVID-19 and non-COVID-19 groups.

Comparison of clinical features between the two groups of patients with and without COVID-19 is reported in Table 2. There is no significant difference in age and sex between the two groups. Significant differences are found in common symptoms between groups, including fever (*p = 0.003*), dry cough (*p = 0.025*), and fatigue (*p = 0.007*). The respiration rate and heart rate also show significant differences between the two groups (both *p < 0.001*). Compared with non-COVID-19 pneumonia, the reduction of the WBC count is more pronounced in COVID-19 patients (*p < 0.001*). The ratio of lymphocyte and ratio of neutrophil also show a significant difference between COVID-19 and non-COVID-19 groups. Although lymphopenia was observed in 32 COVID-19 patients (45.71%), it is not statistically different compared with that in the non-COVID-19 group. C-creative protein (CRP) level and procalcitonin level are also significantly different between the two groups (*p < 0.001* and *p = 0.007*, respectively). Most COVID-19 patients present normal procalcitonin level (82.86%).

### Clinical and radiological feature selection

Of the features, 18 radiological features and 17 clinical features were selected to form the predictors based on the result from Tables 1 and 2. Table 3 lists the features selected by univariate analysis and LASSO.

**Table 3:** Selected features in C, R, and CR models

Model and individual features	Coefficients
R, *n* = 8 (41)*	
Intercept	− 0.307
Total number of mixed GGO in peripheral area	0.359
Total number of consolidation	− 1.262
Total number of solid nodules with ground-glass opacities	0.452
Interlobular septal thickening	− 5.559
Crazy paving pattern	3.566
Tree-in-bud	− 2.548
Pleural thickening	3.265
Offending vessel augmentation in lesions	5.504
C, *n* = 7 (26)*	
Intercept	29.273
Respiration	− 0.359
Heart rate	− 0.054
Temperature	− 0.289
White blood cell count	− 0.175
Cough	− 1.866
Fatigue	2.855
Lymphocyte count category	− 0.028
CR, *n* = 10 (67)*	
Intercept	45.117
Total number of mixed GGO in peripheral area	0.108
Tree-in-bud	− 1.853
Offending vessel augmentation in lesions	6.000
Respiration	− 0.583
Heart ratio	− 0.084
Temperature	− 0.536
White blood cell count	− 0.471
Cough	− 0.997
Fatigue	− 0.228
Lymphocyte count category	− 2.177

C, R, and CR indicate the predicted model based on clinical features, radiological features, and the combination of clinical features and clinical radiological features, respectively

*n means corresponding selected features, and data in parentheses are total features. Coefficients: the estimate value of each feature in multivariate logistic regression model by “glm” package in R

### Model development and validation

**Fig. 3:**
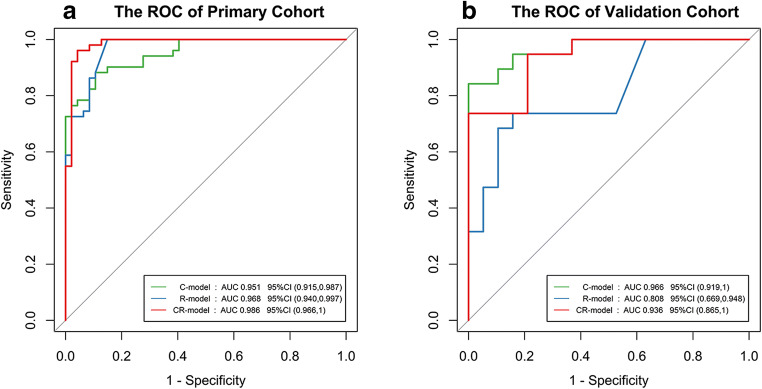
ROC of the three models in primary and validation cohort curves. Comparison of receiver operating characteristic (ROC) curves among the radiological mode (R model), clinical model (C model), and the combination of clinical and radiological model (CR model) for the diagnosis of COVID-19 in the primary (**a**) and validation (**b**) cohorts

The prediction models based on (i) clinical features (C model), (ii) radiological features (R model), and (iii) the combination of clinical features and radiological features (CR model) were developed. ROC analyses for the primary and validation cohort are shown in Table 4 and Fig. 3. The CR model yielded a maximum AUC of 0.986 (95% CI 0.966~1.000) in the primary cohort with the highest accuracy and specificity, which was 0.936 (95% CI 0.866~1.000) in the validation cohort. The AUC for the C model was 0.952 (95% CI 0.988~0.915) and 0.967 (95% CI 0.919~1.000) in the primary and validation cohorts, respectively. For the R model, the AUC of the two cohorts was 0.969 (95% CI 0.940~0.997) and 0.809 (95% CI 0.669~0.948), respectively.

**Table 4:** Performance of the individualized prediction models

	Primary cohort (*n* = 98)					Validation cohort (*n* = 38)				
Models	AUC	95% CI	Accuracy	Specificity	Sensitivity	AUC	95% CI	Accuracy	Specificity	Sensitivity
C model	0.952	0.915~0.988	0.888	0.894	0.882	0.967	0.919~1.000	0.868	0.859	0.842
R model	0.969	0.940~0.997	0.929	0.851	1.000	0.809	0.669~0.948	0.684	0.368	1.000
CR model	0.986	0.966~1.000	0.959	0.957	0.961	0.936	0.866~1.000	0.763	0.789	0.737

C, R, and CR indicate the predicted model based on clinical features, radiological features, and the combination of clinical features and clinical radiological features, respectively. *CI* confidence interval

**Fig. 4:**
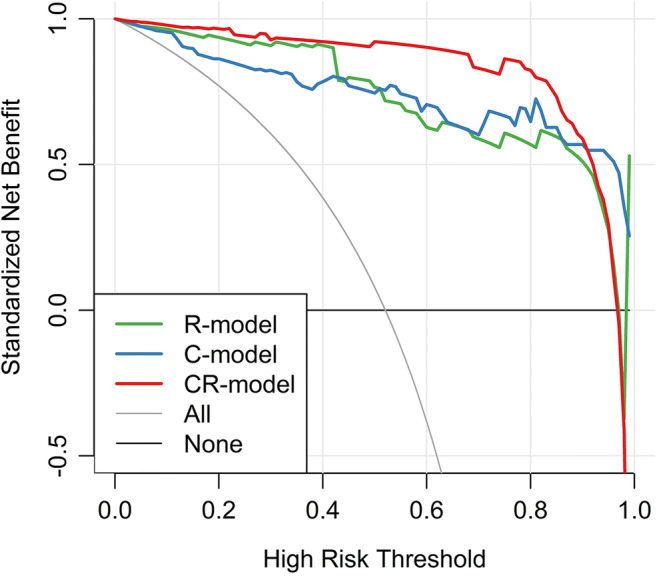
Decision curve analysis for each model in the primary dataset. The *y*-axis measures the net benefit, which is calculated by summing the benefits (true-positive findings) and subtracting the harms (false-positive findings), weighting the latter by a factor related to the relative harm of undetected metastasis compared with the harm of unnecessary treatment. The decision curve shows that if the threshold probability is over 10%, the application of the combination of clinical and radiological model (CR model) to diagnose COVID-19 adds more benefit than the clinical model (C model) and radiological model (R model)

To determine the clinical usefulness of the diagnostic model, we developed the decision curve (Fig. 4), which showed better performances for the CR model compared with that for the C model and the R model. Across the majority of the range of reasonable threshold probabilities, the decision curve analysis showed that the CR model had a higher overall benefit than the C model and R model.

**Fig. 5:**
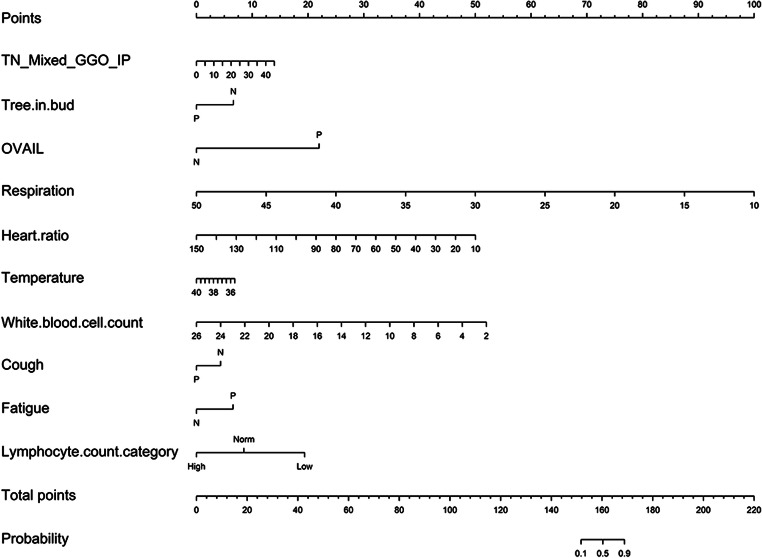
Nomogram of the CR model in the primary cohort. TN_Mixed_GGO_IP represented the total number of mixed GGO in peripheral area. AVAIL represented offending vessel segmentation in lesions. N was a negative result, and P was a positive result. Norm represented normal. Note that in probability scale, 0 = non-COVID-19, 1 = COVID-19

The nomogram (Fig. 5) was developed by the CR model in the primary cohort, with the factors of the total number of mixed GGO in peripheral area (TN_Mixed_GGO_IP), tree-in-bud, offending vessel augmentation in lesions (OVAIL), respiration, heart ratio, temperature, white blood cell count, cough, fatigue and lymphocyte count category incorporated. The total points were calculated by summing the points identified on the “points” scale for each factor. By comparing the “total points” scale and the “probability” scale, the individual probability of COVID-19 infection could be obtained.

## Discussion

In this multi-center study, statistical analysis was performed in comparing imaging and clinical manifestations between pneumonia patients with and without COVID-19. Eighteen radiological semantic features and seventeen clinical features were identified to be significantly different between the two groups (*p < 0.05*). Three models for COVID-19 diagnosis were developed based on the refined features. The models were validated in the both primary and validation cohorts and achieved an AUC as high as 0.986. These models will play an essential role for early and easy-to-access diagnosis, especially when there are not enough RT-PCT kits or experimental platforms to test for the COVID-19 infection.

A total of 1745 lesions were evaluated for the qualitative feature, location, and size in this study. Consistent with the previous studies, the ground-glass opacities and consolidation in the lung periphery were considered to be the imaging hallmark in patients with COVID-19 infection [11, 25]. However, when we subdivided the GGO into pure GGO and mixed GGO, we found that the distribution pattern is different between these two lesions. Pure GGO show differences between groups in every location of the lungs, whereas mixed GGO only have significant differences between groups in the lung periphery. Recent studies defined four stages of lung involvement in COVID-19 [26]. Therefore, a follow-up analysis of these distributions would be significant. The lesion size in patients with COVID-19 infection was another interesting observation. Most lesions were between 1 and 3 cm, with few lesions larger than half of the lung segment, which was similar to the finding in MERS_CoV [22]. Other features similar to MERS_CoV and SARS_CoV were observed in the laboratory abnormalities, such as lymphopenia, which may be associated with the cellular immune deficiency [3, 27]. However, our results showed no significant difference in lymphopenia between the COVID-19 and non-COVID-19 patients.

To our knowledge, no diagnostic model based on imaging and clinical features alone has been proposed for the diagnosis of COVID-19. Our clinical and radiological semantic (CR) models consisted of the following features: total number of GGO with consolidation in the peripheral area, tree-in-bud, offending vessel augmentation in lesions, temperature, heart ratio, respiration, cough and fatigue, WBC count, and lymphocyte count category. The CR model outperformed the individual clinical and radiologic model. This result was in accordance with that in previous study in breast cancer, in which the model based on the combination of radiomics features and clinical features achieved a higher performance [24]. Compared with the radiomics-based model, the extraction of radiological semantic features can overcome the image discrepancy caused by different scanning parameters and/or different CT vendors. A previous study [28] also indicated that models based on semantic features determined by an experienced thoracic radiologist slightly outperformed models based on computed texture features alone.

There are a few limitations in this study. First, the sample size is relatively small because this is a retrospective analysis of a new disease and most of the cases outside of Wuhan City are imported. Second, with the multi-center retrospective design, there is a potential bias of patient selection [29], since there may be some deviations in marking semantic features among readers, though we have taken the effort to reduce this by creating pictorial examples and setting feature criteria (Supplementary Materials). Third, longitudinal CT study was not performed. Whether or not this model can be used to evaluate the follow-ups and help to guide therapy remains an open question to be further explored. Moreover, the rich high-order features of the CT image combined with radiomics or deep learning have not been studied, which may be another way to identify the patients with COVID-19. Besides, one can also focus on the role of radiological features in disease monitoring, treatment evaluation, and prognosis prediction.

In conclusion, 1745 lesions and 67 features were compared between pneumonia patients with and without COVID-19. Thirty-five features were significantly different between the two groups. A diagnostic model with AUC as high as 0.986 was developed and validated both in the primary and in the validation cohorts, which may help improve the COVID-19 diagnosis.

## Electronic supplementary material

ESM 1 (DOCX 404 kb)

## Abbreviations

AUC: Area under the curve
C model: Clinical features model
COVID-19: Coronavirus disease 2019
GGO: Ground-glass opacities
MERS-CoV: Middle East respiratory syndrome coronavirus
R model: Radiological semantic features model
ROC: Receiver operating characteristic
RT-PCR: Reverse transcription polymerase chain reaction
SARS-Cov: Severe acute respiratory syndrome coronavirus
SARS-CoV-2: Severe acute respiratory syndrome coronavirus 2
