# Supplementary material for: A diagnostic model for coronavirus disease 2019 (COVID-19) based on radiological semantic and clinical features: a multi-center study
Source: Eur Radiol. 2020 Apr 16;30(9):4893–902. doi: 10.1007/s00330-020-06829-2 (PMC7160614; doi:10.1007/s00330-020-06829-2)
Supplement: Supplementary file 1 — (DOCX 404 kb) [file 330_2020_6829_MOESM1_ESM.docx]

**Supplementary Materials**

**Table of Contents：**

[**1. Patient selection to Meizhou people’s hospital (Figure E1) 2**](#_Toc35425435)

[**2. CT imaging protocols and acquisition parameters (Table E1) 3**](#_Toc35425436)

[**3. Definition of radiological semantic features (Table E2) 5**](#_Toc35425437)

[**4. The flow chart of features selection process for three models (Figure E2) 7**](#_Toc35425438)

[**5. Decision curve analysis 8**](#_Toc35425439)

[**6. Calibration curves of the CR-model (Figure E3) 9**](#_Toc35425440)

[**7. Supplementary radiological semantic features of patients in COVID-19 and non-COVID-19 (Table E3) 10**](#_Toc35425441)

[**8. Supplementary radiological sign of patients in COVID-19 and non-COVID-19 (Table E4) 12**](#_Toc35425442)

[**9. Supplementary radiological features of patients in primary cohort and validation cohort (Table E5) 13**](#_Toc35425443)

[**10. Supplementary Clinical features of patients in primary cohort and validation cohort (Table E6) 16**](#_Toc35425444)

# Patient selection to Meizhou people’s hospital (Figure E1)


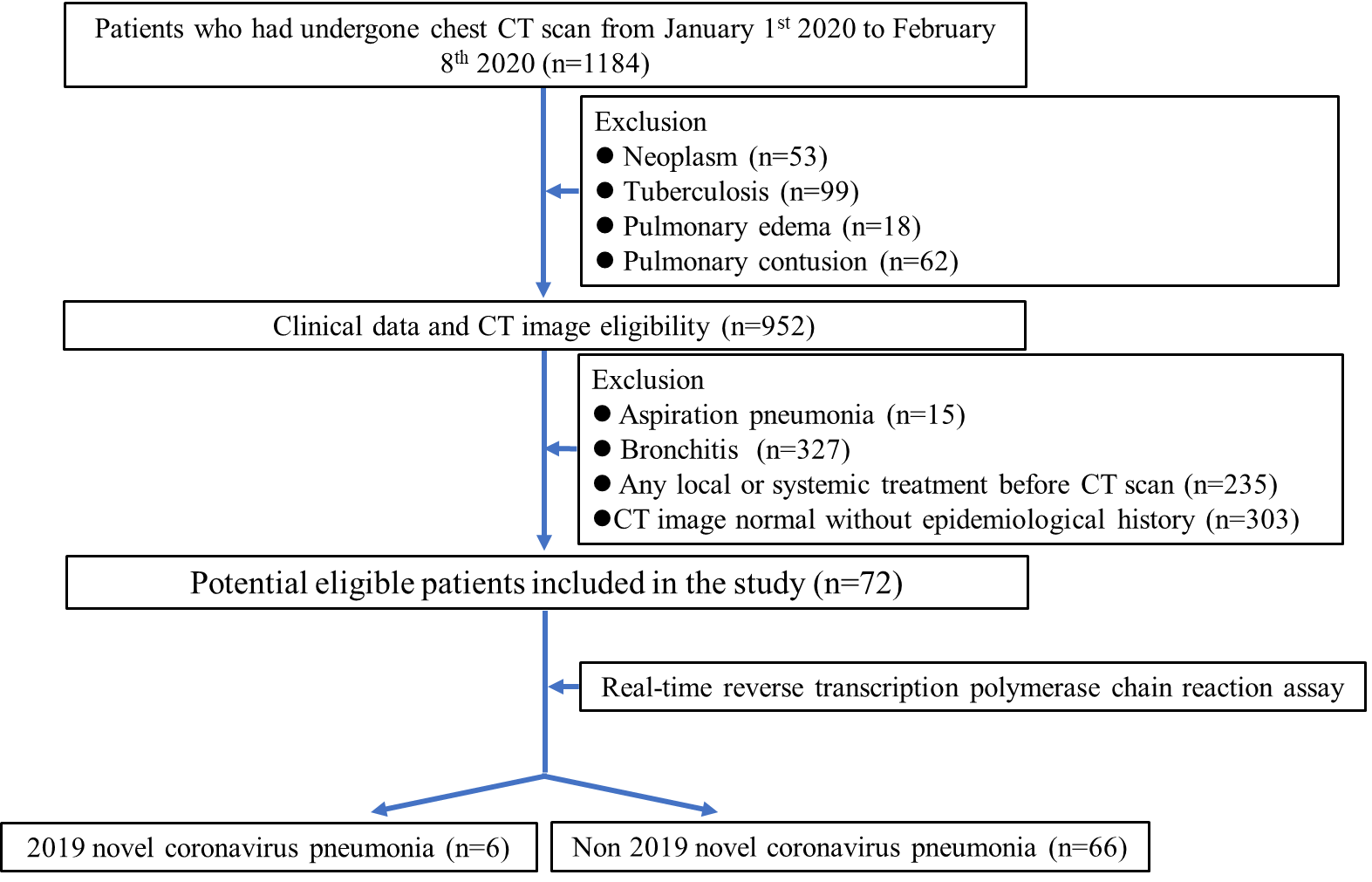


Figure E1: Flowchart shows patient selection to Meizhou people’s hospital.

# CT imaging protocols and acquisition parameters (Table E1)

We selected patient images that were acquired on various models of multi-row spiral CT scans from GE, Scenariaand Philips scanners. Table E1 shows the CT imaging protocols and acquisition parameters of each medical institutions.

Table E1: CT imaging protocols and acquisition parameters of each medical institutions

| CT imaging protocols and acquisition parameters | Yongzhou people's hospital, Hunan province | The first affiliated hospital of Shantou university medical college | Shantou central hospital, Guangdong province | Huizhou central hospital, Guangdong province | Meizhou people’s hospital, Guangdong province |
| --- | --- | --- | --- | --- | --- |
| CT scanner | 64-section scanner (SCENARIA 64 CT, Hitachi Medical). | 64-section scanner (GE CT Discovery 750 HD) | 64-section scanner (Ingenuity CT, PHILIPS) | 64-section scanner (Ingenuity CT, PHILIPS) | 64-slice multidetector spiral CT (Somatom Definition AS, Siemens) |
| Tube voltage (kV) | 130 | 120 | 120 | 120 | 120 |
| Automatic tube current (mA) | 180-350 | 320 | 132-459 | 400 -500 | 150 |
| Iterative reconstruction technique | + | + | + | + | + |
| Detector (mm) | 64 | 40 | 64 | 64 | 128 |
| Rotation time (s) | 0.5 | 0.5 | 0.75 | 0.5 | 0.5 |
| Section thickness (mm) | 5 | 5 | 5 | 5 | 1.5 |
| Collimation | 0.6 | 0.6 | 0.625 | 0.625 | 0.6 |
| Pitch | 0.99 | 1.5 | 0.98 | 0.9 | 1.2 |
| Matrix | 512 × 512 | 512 × 512 | 512 × 512 | 512 × 512 | 512 × 512 |

# Definition of radiological semantic features (Table E2)

Table E2 presents definitions of radiological feature, which could benefit to understand each feature in CT images and read smoothly. These definitions are cited from study of Hansell DM, et al in 2008 (1).

Table E2: definition of radiological feature.

| Radiological Feature | Definition |
| --- | --- |
| Ground-glass opacities(GGO) | It appears as hazy increased opacity of lung, with preservation of bronchial and vascular margins. |
| Consolidation | Consolidation appears as a homogeneous increase in pulmonary parenchymal attenuation that obscures the margins of vessels and airway walls. |
| Pure GGO | Equal to GGO |
| Mixed GGO | The combination of GGO and consolidation |
| Emphysema | The CT appearance of emphysema consists of focal areas or regions of low attenuation, usually without visible walls. |
| Air bronchogram | An air bronchogram is a pattern of air-filled (low-attenuation) bronchi on a back-ground of opaque (high-attenuation) air-less lung. |
| Interlobular septal thickening | On CT scans, disease affecting one of the components of the septa may be responsible for thickening and so render septa visible. |
| Crazy-paving pattern | This pattern appears as thickened interlobular septa and intralobular lines superimposed on a background of ground-glass opacity, resembling irregularly shaped paving stones. |
| Tree-in-bud sign | The tree-in-bud pattern represents centrilobular branching structures that resemble a budding tree. |
| Cavity | A cavity is a gas-filled space, seen as a lucency or low-attenuation area, within pulmonary consolidation, a mass, or a nodule. |
| Lymphadenopathy | By common usage, the term lymphadenopathy is usually restricted to enlargement, due to any cause, of the lymph nodes. Somewhat arbitrary thresholds for the upper limit of normal of 1 cm in short-axis diameter for mediastinal nodes and 3 mm for most hilar nodes have been reported. |
| Offending vessel augmentation in lesions | The vessel associated with lesions is enlarged in CT image. |
| Pleural thickening | The involved pleura is thickening as elevated flat or nodular lesions. |

1. **The flow chart of features selection process for three models (Figure E2)**

**
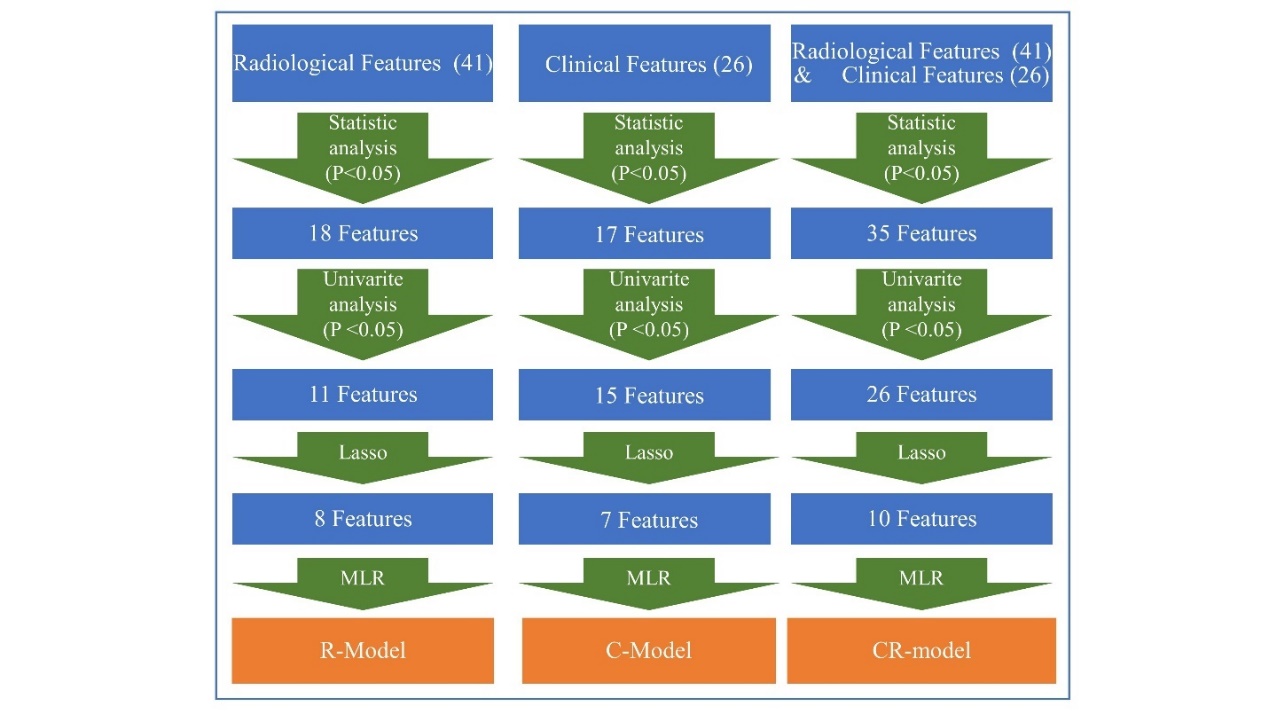
**

Figure E2. The flow chart of features selection process for three models, including C-model, R-model and CR-model . LASSO is least absolute shrinkage and selection operator. MLR is multivariate logistic regression.

1. **Decision curve analysis**

Decision curve analysis is a plot of the “net benefit” against “threshold probabilities”. The y-axis measures the net benefit (NB) which is calculated by subtracting the proportion of all patients who are false positive (B) from the proportion who are true positive (A), weighting by the relative harm (Pt/(1-Pt)) of forgoing treatment compared with the negative consequences of unnecessary treatment. The equation is represented as follows:

NB = A – B × Pt / (1 – Pt) (1)

Pt in equation (1) is represents the threshold probability, where the expected benefit of treatment is equal to the expected benefit of avoiding treatment, i.e.

(b-d) / (a-c)= Pt /(1–Pt) (2)

where (a-c) is the harm from a false-negative result; (b-d) is the harm from a false-positive result. a, b, c and d represent the value of true positive, false positive, false negative, and true negative, respectively. At which time a patient will opt for treatment informs us of how a patient weighs the relative harms of false-positive results and false-negative results.

1. **Calibration curves of the CR-model (Figure E3)**

***
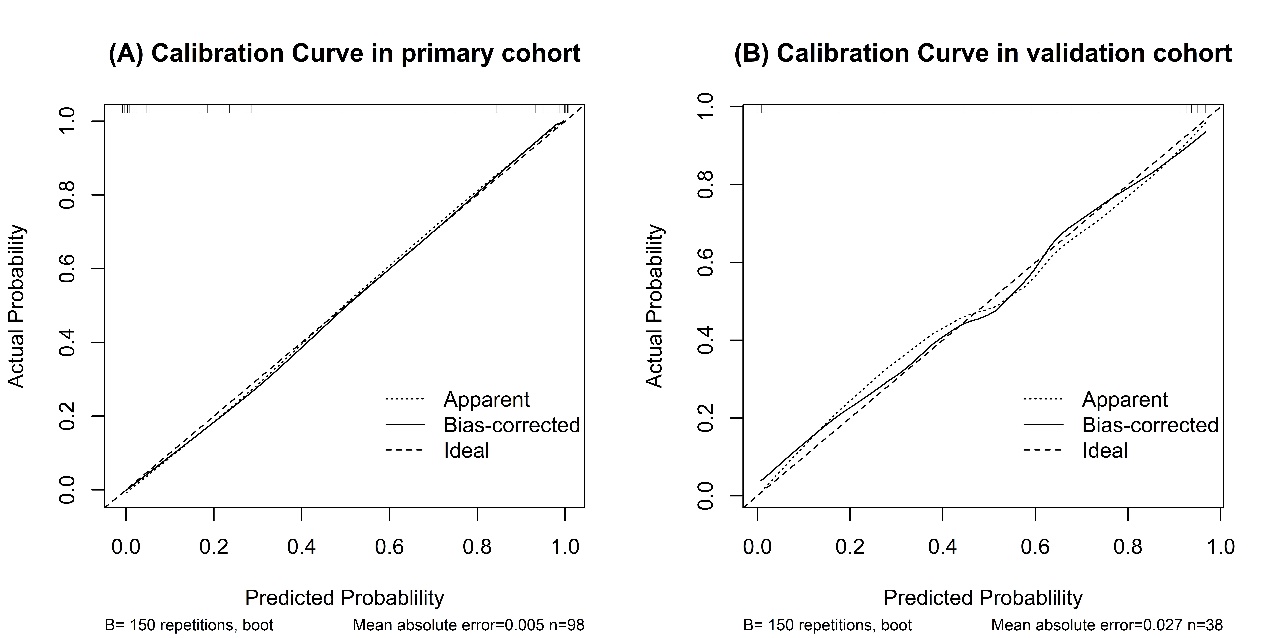
***

Figure E3. Calibration curves of the CR-model in the primary cohort (A) and validation cohort (B).

# Supplementary radiological semantic features of patients in COVID-19 and non-COVID-19 (Table E3)

|  | Table E3: Supplementary radiological semantic features of patients in COVID-19 and non-COVID-19 | | | |  |
| --- | --- | --- | --- | --- | --- |
|  | Feature | non-COVID-19  (*n*=66) | COVID-19  (*n*=70) | *P*-value |  |
|  | Overall condition of lesions |  |  | 0.045^c^* |  |
|  | Normal | 0(0.00 %) | 7(10.00 %) |  |  |
|  | Single | 5(7.58 %) | 6(8.57 %) |  |  |
|  | Multiple | 61(92.42 %) | 57(81.43 %) |  |  |
|  | Lesion sizes |  |  |  |  |
|  | < 1cm^#^ | 0.00(0.00, 4.00) | 1.00(0.00, 4.05) | 0.072^b^ |  |
|  | 1cm to 3cm^#^ | 2.00(0.00, 7.00) | 4.00(1.00, 10.00) | 0.027^b^* |  |
|  | 3cm to half of segments^#^ | 0.00(0.00, 1.00) | 0.50(0.00, 3.00) | 0.141^b^ |  |
|  | ＞half of segments^#^ | 1.00(0.00, 2.00) | 0.00(0.00, 1.00) | 0.017^b^* |  |
|  | Total scores of involved lung zones |  |  |  |  |
|  | Bilateral lung^#^ | 5.00(2.95, 7.00) | 4.50(2.00, 8.00) | 0.417^b^ |  |
|  | Right lung^#^ | 3.00(1.00, 4.05) | 3.00(1.00, 5.00) | 0.802^b^ |  |
|  | Left lung^#^ | 2.00(1.00, 3.00) | 2.00(1.00, 3.00) | 0.445^b^ |  |
|  | Bilateral upper lobes^#^ | 2.00(0.00, 3.00) | 2.00(0.00, 2.00) | 0.724^b^ |  |
|  | Bilateral lower lobes^#^ | 2.00(1.95, 3.05) | 2.00(1.00, 4.00) | 0.655^b^ |  |
|  | Number of lobes affected |  |  |  |  |
|  | Right lung^#^ | 2.00(1.00, 3.00) | 2.00(1.00, 3.00) | 0.943^b^ |  |
|  | Left lung^#^ | 2.00(1.00, 2.00) | 2.00(1.00, 2.00) | 0.663^b^ |  |
|  | Total number of lobes affected in Bilateral lung |  |  |  |  |
|  | ＜3 lobes | 26(39.39%) | 24(34.29%) | 0.537^a^ |  |
|  | ≥ 3 lobes | 40(60.61%) | 46(65.71%) |  |  |
|  | Total number of involved lung segments | | | |  |
|  | Bilateral lung^#^ | 4.00(2.00, 5.00) | 4.00(2.00, 5.00) | 0.660^b^ |  |
|  | Bilateral upper lobes^#^ | 1.00(0.00, 2.00) | 2.00(0.00, 2.00) | 0.391^b^ |  |
|  | Bilateral lower lobes^#^ | 2.00(1.00, 2.00) | 2.00(1.00, 2.00) | 0.516^b^ |  |
|  | Note.- * Data with statistical significance. *P*^a^: chi square test, *P*^b^: student’s *t* test, *P*^c^ Kruskal-Wallis *H*-test.  # Results are median with interquartile range in parentheses, and the remainder results are measurements with corresponding ratio in parentheses. | | | |  |

# Supplementary radiological sign of patients in COVID-19 and non-COVID-19 (Table E4)

|  | Table E4: Supplementary radiological sign of patients in COVID-19 and non-COVID-19 | | | |  |
| --- | --- | --- | --- | --- | --- |
|  | Radiological sign | non-COVID-19  (*n*=66) | COVID-19  (*n*=70) | *P*-value |  |
|  | Pleural traction sign |  |  |  |  |
|  | Negative | 46(69.70%) | 35(50.00%) | 0.019^a^* |  |
|  | Positive | 20(30.30%) | 35(50.00%) |  |  |
|  | Linear opacities |  |  |  |  |
|  | Negative | 34(51.52%) | 30(42.86%) | 0.312^a^ |  |
|  | Positive | 32(48.48%) | 40(57.14%) |  |  |
|  | Emphysema |  |  |  |  |
|  | Negative | 60(90.91%) | 64(91.43%) | 0.915^a^ |  |
|  | Positive | 6(9.09%) | 6(8.57%) |  |  |
|  | Air bronchogram |  |  |  |  |
|  | Negative | 22(33.33%) | 28(40.00%) | 0.420^a^ |  |
|  | Positive | 44(66.67%) | 42(60.00%) |  |  |
|  | Bronchial wall thickening |  |  |  |  |
|  | Negative | 49(74.24%) | 28(40.00%) | <0.001^a^* |  |
|  | Positive | 17(25.76%) | 42(60.00%) |  |  |
|  | Pleural Effusions |  |  |  |  |
|  | Negative | 46(69.70%) | 70(100.00%) | <0.001^a^* |  |
|  | Positive | 20(30.30%) | 0(0.00%) |  |  |
|  | Cavity |  |  |  |  |
|  | Negative | 66(100.00%) | 68(97.14%) | 0.497^a^ |  |
|  | Positive | 0(0.00%) | 2(2.86%) |  |  |
|  | Lymphadenopathy |  |  |  |  |
|  | Negative | 65(98.48%) | 70(100.00%) | 0.485^a^ |  |
|  | Positive | 1(1.52%) | 0(0.00%) |  |  |
|  | Note.- * Data with statistical significance. *P*^a^: chi square test.  # Results are measurements with corresponding ratio in parentheses. | | | |  |

1. **Supplementary radiological features of patients in primary cohort and validation cohort (Table E5)**

|  | Table E5: Radiological semantic features of patients in primary and validation cohort | | | |  |
| --- | --- | --- | --- | --- | --- |
|  | Feature | primary cohort  (*n*=98) | validation cohort (*n*=38) | *P*-value |  |
|  | Number of pure GGO |  |  |  |  |
|  | Total^#^ | 2.50(0.00, 8.00) | 1.00(0.00, 5.05) | 0.243^b^ |  |
|  | Peripheral area^#^ | 2.00(0.00, 7.00) | 1.00(0.00, 4.00) | 0.242^b^ |  |
|  | Central / both peripheral and central area^#^ | 0.00(0.00, 1.00) | 0.00(0.00, 0.00) | 0.341^b^ |  |
|  | Number of mixed GGO |  |  |  |  |
|  | Total^#^ | 3.00(1.00, 6.00) | 1.00(0.00, 5.05) | 0.085^b^ |  |
|  | Peripheral area^#^ | 1.00(0.00, 4.00) | 0.50(0.00, 5.00) | 0.241^b^ |  |
|  | Central / both peripheral and central area^#^ | 1.00(0.00, 2.00) | 0.00(0.00, 1.00) | 0.038^b^* |  |
|  | Total number of consolidation |  |  |  |  |
|  | Consolidation^#^ | 0.00(0.00, 2.00) | 0.00(0.00, 1.00) | 0.081^b^ |  |
|  | Pure solid nodules^#^ | 0.00(0.00, 0.00) | 0.00(0.00, 0.00) | 0.331^b^ |  |
|  | Solid nodules with GGO^#^ | 0.00(0.00, 1.00) | 0.00(0.00, 0.00) | 0.282^b^ |  |
|  | Total number of lesions |  |  |  |  |
|  | Peripheral area^#^ | 7.00(3.00, 12.00) | 4.00(1.00, 8.05) | 0.021^b^* |  |
|  | Central area ^#^ | 0.00(0.00, 2.00) | 0.00(0.00, 1.00) | 0.272^b^ |  |
|  | Both peripheral and central area^#^ | 1.00(0.00, 2.00) | 0.00(0.00, 1.00) | 0.085^b^ |  |
|  | Interlobular septal thickening |  |  |  |  |
|  | Negative | 49(50.00%) | 26(68.42%) | 0.053^a^ |  |
|  | Positive | 49(50.00%) | 12(31.58%) |  |  |
|  | Crazy-paving pattern |  |  |  |  |
|  | Negative | 62(63.27%) | 30(78.95%) | 0.079^a^ |  |
|  | Positive | 36(36.73%) | 8(21.05%) |  |  |
|  | Tree-in-bud |  |  |  |  |
|  | Negative | 66(67.35%) | 32(84.21%) | 0.050^a^ |  |
|  | Positive | 32(32.65%) | 6(15.79%) |  |  |
|  | Pleural thickening |  |  |  |  |
|  | Negative | 59(60.20%) | 23(60.53%) | 0.973^a^ |  |
|  | Positive | 39(39.80%) | 15(39.47%) |  |  |
|  | Offending vessel augmentation in lesions | |  |  |  |
|  | Negative | 56(57.14%) | 16(42.11%) | 0.115^a^ |  |
|  | Positive | 42(42.86%) | 22(57.89%) |  |  |
|  | Overall condition of lesions |  |  | 0.041^c^* |  |
|  | Normal | 6(6.12 %) | 1(2.63 %) |  |  |
|  | Single | 3(3.06 %) | 8(21.05 %) |  |  |
|  | Multiple | 89(90.82 %) | 29(76.32 %) |  |  |
|  | Lesion sizes |  |  |  |  |
|  | < 1cm^#^ | 1.00(0.00, 5.00) | 0.00(0.00, 3.05) | 0.441^b^ |  |
|  | 1cm to 3cm^#^ | 3.50(0.95, 9.00) | 1.00(0.00, 6.00) | 0.096^b^ |  |
|  | 3cm to half of segments^#^ | 0.00(0.00, 2.00) | 1.00(0.00, 1.05) | 0.979^b^ |  |
|  | ＞half of segments^#^ | 1.00(0.00, 2.00) | 0.00(0.00, 1.00) | 0.055^b^ |  |
|  | Total scores of involved lung zones | |  |  |  |
|  | Bilateral lung^#^ | 5.00(3.00, 8.00) | 3.00(1.00, 6.00) | 0.008^b^* |  |
|  | Right lung^#^ | 3.00(1.95, 5.00) | 1.00(0.95, 4.00) | 0.010^b^* |  |
|  | Left lung^#^ | 2.00(1.00, 3.00) | 2.00(1.00, 2.05) | 0.102^b^ |  |
|  | Bilateral upper lobes^#^ | 2.00(1.00, 3.00) | 1.00(0.00, 2.00) | 0.039^b^* |  |
|  | Bilateral lower lobes^#^ | 2.00(2.00, 4.00) | 2.00(1.00, 3.00) | 0.054^b^ |  |
|  | Number of lobes affected |  |  |  |  |
|  | Right lung^#^ | 3.00(1.00, 3.00) | 1.00(0.00, 2.05) | 0.003^b^* |  |
|  | Left lung^#^ | 2.00(1.00, 2.00) | 1.00(1.00, 2.00) | 0.263^b^ |  |
|  | Total number of lobes affected in Bilateral lung | |  |  |  |
|  | ＜3 lobes | 30(30.61%) | 20(52.63%) | 0.017^a^* |  |
|  | ≥ 3 lobes | 68(69.39%) | 18(47.37%) |  |  |
|  | Total number of involved lung segments | | | |  |
|  | Bilateral lung^#^ | 5.00(2.00, 5.00) | 2.00(1.00, 4.05) | 0.008^b^* |  |
|  | Bilateral upper lobes^#^ | 2.00(0.95, 2.00) | 1.00(0.00, 2.00) | 0.120^b^ |  |
|  | Bilateral lower lobes^#^ | 2.00(1.00, 2.00) | 1.00(1.00, 2.00) | 0.061^b^ |  |
|  | Pleural traction sign |  |  |  |  |
|  | Negative | 58(59.18%) | 23(60.53%) | 0.886^a^ |  |
|  | Positive | 40(40.82%) | 15(39.47%) |  |  |
|  | Linear opacities |  |  |  |  |
|  | Negative | 46(46.94%) | 18(47.37%) | 0.964^a^ |  |
|  | Positive | 52(53.06%) | 20(52.63%) |  |  |
|  | Emphysema |  |  |  |  |
|  | Negative | 88(89.80%) | 36(94.74%) | 0.566^a^ |  |
|  | Positive | 10(10.20%) | 2(5.26%) |  |  |
|  | Air bronchogram |  |  |  |  |
|  | Negative | 33(33.67%) | 17(44.74%) | 0.230^a^ |  |
|  | Positive | 65(66.33%) | 21(55.26%) |  |  |
|  | Bronchial wall thickening |  |  |  |  |
|  | Negative | 52(53.06%) | 25(65.79%) | 0.179^a^ |  |
|  | Positive | 46(46.94%) | 13(34.21%) |  |  |
|  | Pleural Effusions |  |  |  |  |
|  | Negative | 81(82.65%) | 35(92.11%) | 0.163^a^ |  |
|  | Positive | 17(17.35%) | 3(7.89%) |  |  |
|  | Cavity |  |  |  |  |
|  | Negative | 97(98.98%) | 37(97.37%) | 0.482^a^ |  |
|  | Positive | 1(1.02%) | 1(2.63%) |  |  |
|  | Lymphadenopathy |  |  |  |  |
|  | Negative | 97(98.98%) | 38(100.00%) | 1.000^a^ |  |
|  | Positive | 1(1.02%) | 0(0.00%) |  |  |
|  | Note.- * Data with statistical significance. ^#^ Results are median with interquartile range in parentheses, and the remainder results are measurements with corresponding ratio in parentheses. *P*^a^: chi square test, *P*^b^: student’s *t* test, *P*^c^: Kruskal-Wallis *H* test. | | | |  |

1. **Supplementary Clinical features of patients in primary cohort and validation cohort (Table E6)**

|  | Table E6: Clinical features of patients in primary and validation cohort | | | |  |
| --- | --- | --- | --- | --- | --- |
|  | Feature | primary cohort (*n*=98) | validation cohort  (*n*=38) | *P*-value |  |
|  | Sex |  |  |  |  |
|  | Male^#^ | 61(62.24%) | 23(60.53%) | 0.853^a^ |  |
|  | Female^#^ | 37(37.76%) | 15(39.47%) |  |  |
|  | Age (years) | 46.21±19.32 | 41.08±21.07 | 0.178^b^ |  |
|  | Vital signs |  |  |  |  |
|  | Systolic blood pressure (mm Hg) | 127.28±18.29 | 126.29±22.05 | 0.791^b^ |  |
|  | Diastolic blood pressure (mm Hg) | 77.96±10.54 | 82.05±18.51 | 0.205^b^ |  |
|  | Respiration rate (bpm) | 22.36±4.89 | 22.68±7.98 | 0.772^b^ |  |
|  | Heart rate (bpm) | 92.63±19.59 | 96.08±16.33 | 0.338^b^ |  |
|  | Temperature (℃) | 37.42±1.04 | 37.22±0.78 | 0.298^b^ |  |
|  | Signs |  |  |  |  |
|  | Dry cough^#^ | 73(74.49%) | 31(81.58%) | 0.382^a^ |  |
|  | Fatigue^#^ | 27(27.55%) | 3(7.89%) | 0.013^a^* |  |
|  | Sore throat^#^ | 11(11.22%) | 4(10.53%) | 0.851^a^ |  |
|  | Stuffy^#^ | 2(2.04%) | 4(10.53%) | 0.090^a^ |  |
|  | Runny nose^#^ | 3(3.06%) | 3(7.89%) | 0.443^a^ |  |
|  | White blood cell  count (× 10^9^/L) | 7.50±4.36 | 10.31±6.34 | 0.015^b^* |  |
|  | Lymphocyte count (× 10^9^/L) | 1.32±0.80 | 1.63±1.53 | 0.246^b^ |  |
|  | Neutrophil count (× 10^9^/L) | 5.54±4.18 | 7.79±5.33 | 0.023^b^* |  |
|  | C-reactive protein (mg/L) | 48.62±56.03 | 43.56±53.44 | 0.633^b^ |  |
|  | Procalcitonin (ng/mL) | 2.00±7.05 | 1.16±4.54 | 0.495^b^ |  |
|  | * Data with statistical significance. *P*^a^: chi square test, *P*^b^: student’s *t* test.  ^#^ Results are measurements with corresponding ratio in parentheses. | | | |  |

Reference

1. Hansell DM, Bankier AA, MacMahon H, McLoud TC, Müller NL, Remy J. Fleischner Society: glossary of terms for thoracic imaging. Radiology. 2008; 246(3):697-722.
